# 05. Pragmatic Assessment of Influenza Vaccine Effectiveness in the DoD (PAIVED): Updates from Year 3 of Multi-Site Trial

**DOI:** 10.1093/ofid/ofab466.208

**Published:** 2021-12-04

**Authors:** Timothy Burgess, Stephanie A Richard, Limone Collins, Rhonda E Colombo, Anuradha Ganesan, Casey Geaney, David Hrncir, Tahaniyat Lalani, Ana E Markelz, Ryan C Maves, Ryan C Maves, Bruce McClenathan, Katrin Mende, Jitu Modi, Jay R Montgomery, Christina Schofield, Srihari Seshadri, Catherine Skerrett, Christina Spooner, Gregory Utz, Tyler Warkentien, Alan Williams, Christian L Coles

**Affiliations:** 1 Infectious Disease Clinical Research Program, Bethesda, Maryland; 2 Infectious Disease Clinical Research Program, Department of Preventive Medicine and Biostatistics, Uniformed Services University of the Health Sciences, Bethesda, MD and Henry M. Jackson Foundation, Bethesda, MD, Bethesda, Maryland; 3 Immunization Health Branch, Defense Health Agency Bethesda, MD, Falls Church, VA, San Diego, CA, Falls Church, VA; 4 Madigan Army Medical Center, Tacoma, WA, Infectious Disease Clinical Research Program, Bethesda, MD, and Henry M. Jackson Foundation for the Advancement of Military Medicine, Inc., Bethesda, MD, Tacoma, Washington; 5 Infectious Disease Clinical Research Program and the Henry M. Jackson Foundation for the Advancement of Military Medicine and Walter Reed National Military Medical Center, Bethesda, MD; 6 Walter Reed National Military Medical Center, Bethesda, MD; 7 Lackland Air Force Base & Carl R. Darnall Army Medical Center, San Antonio, Texas; 8 Infectious Disease Clinical Research Program, Bethesda, MD, The Henry M. Jackson Foundation, Bethesda, MD, and Naval Medical Center Portsmouth, VA, Portsmouth, Virginia; 9 Brooke Army Medical Center, Fort Sam Houston, Texas; 10 Naval Medical Center San Diego, San Diego, CA and Infectious Disease Clinical Research Program, Bethesda, MD, San DIego, California; 11 Womack Army Medical Center, Fort Bragg, NC 28310, Fort Bragg, North Carolina; 12 Infectious Disease Clinical Research Program, Bethesda, MD, The Henry M. Jackson Foundation, Bethesda, MD, and Brooke Army Medical Center, Fort Sam Houston, TX, San Antonio, TX; 13 Naval Health Clinic Annapolis, Laurel, Maryland; 14 Defense Health Agency, Vienna, Virginia; 15 Madigan Army Medical Center, Tacoma, WA, Tacoma, Washington; 16 Immunization Health Branch, Defense Health Agency, Falls Church, VA; 17 Lackland Air Force Base, San Antonio, Texas; 18 Uniformed Services University of the Health Sciences, Bethesda, Maryland; 19 Naval Medical Center Portsmouth, Portsmouth, VA, Portsmouth, VA; 20 USUHS/FAM, Bethesda, Maryland; 21 Infectious Disease Clinical Research Program, Bethesda, MD, The Henry M. Jackson Foundation, Bethesda, MD, Bethesda, MD

## Abstract

**Background:**

The SARS-CoV-2 pandemic has spotlighted respiratory infections and the value of effective vaccines. The SARS-CoV-2 vaccine has been remarkably effective; however, influenza vaccine effectiveness has been reported to be lower among active duty military populations than in the general public (18% vs 36%). The Pragmatic Assessment of Influenza Vaccine Effectiveness in the DoD (PAIVED) study compares 3 FDA-licensed influenza vaccine types (egg-based, cell-based, and recombinant) to assess differences in immunogenicity and effectiveness in adults.

**Methods:**

Participants in the 3rd year of PAIVED (2020/21 influenza season) were enrolled from October 2020 through January 2021. Participants received weekly surveys about influenza-like-illnesses (ILI) experienced in the past week; if they reported an ILI, they were queried about symptom duration and severity, and asked to self-collect a nasal swab and dried blood sample. Four weeks later, more information about symptom duration and illness burden was obtained via telephone interview, and the participant collected a second blood sample.

**Results:**

PAIVED year 3 enrolled 3,269 participants (Table 1). 278 participants reported 1 ILI , while 60 reported 2 ILIs, and 18 reported 3 ILIs. No pathogen was identified for most processed ILI samples (78%); the most common viruses were SARS-CoV-2 (25, 12%), rhinovirus (24, 12%), and seasonal coronaviruses (4, 2%). No influenza has been identified thus far. Among those participants who had convalescent ILI visits (275), the median duration of the reported ILIs was 9 days (IQR 5, 15), with a median of 4 days (IQR 2, 7) of limited activity, and 2 days (IQR 0, 3) with fever. Three individuals were hospitalized.

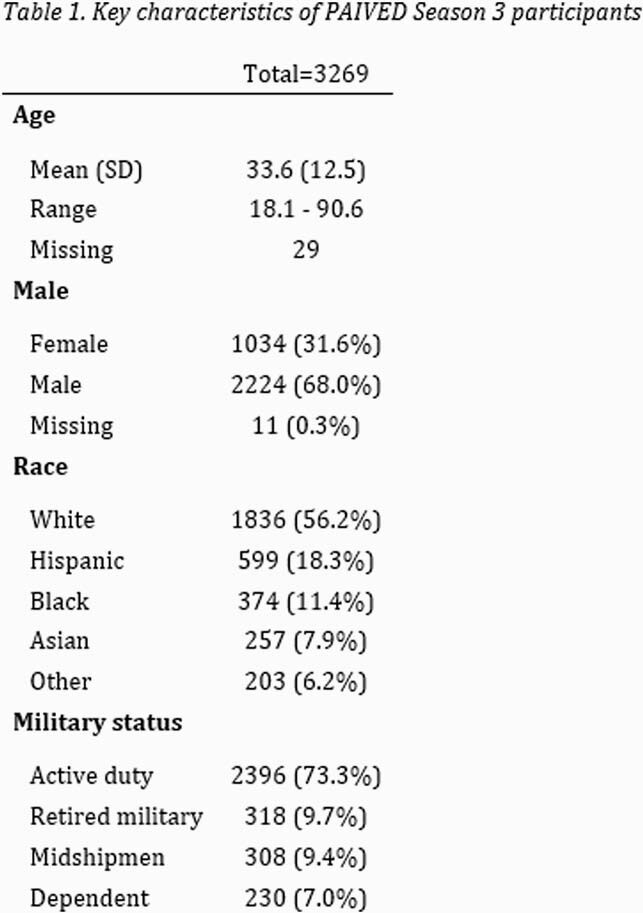

**Conclusion:**

There have been relatively low rates of ILI identified in this study during this season, with only 11% of the participants reporting an ILI so far, consistent with low rates of non-COVID-19 ILI reported elsewhere during the current pandemic. We anticipate some influenza cases may be identified as more samples are processed. Planned analyses include calculating comparative influenza vaccine effectiveness to inform future vaccine purchasing decisions, as well as comparing serological response to the different vaccines.

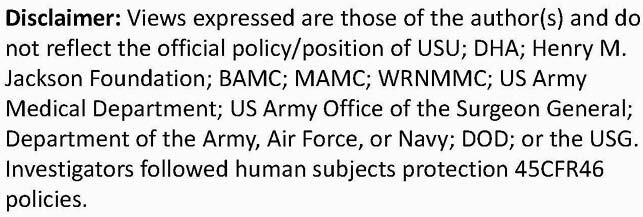

**Disclosures:**

**Ryan C. Maves, MD**, **EMD Serono** (Advisor or Review Panel member)**Heron Therapeutics** (Advisor or Review Panel member) **Jitu Modi, MD**, **GSK** (Speaker’s Bureau)

